# LigandDiff: de Novo Ligand Design for 3D Transition Metal Complexes with Diffusion Models

**DOI:** 10.1021/acs.jctc.4c00232

**Published:** 2024-05-14

**Authors:** Hongni Jin, Kenneth M. Merz

**Affiliations:** † Department of Chemistry, Michigan State University, East Lansing, Michigan 48824, United States; ‡ Department of Biochemistry and Molecular Biology, Michigan State University, East Lansing, Michigan 48824, United States

## Abstract

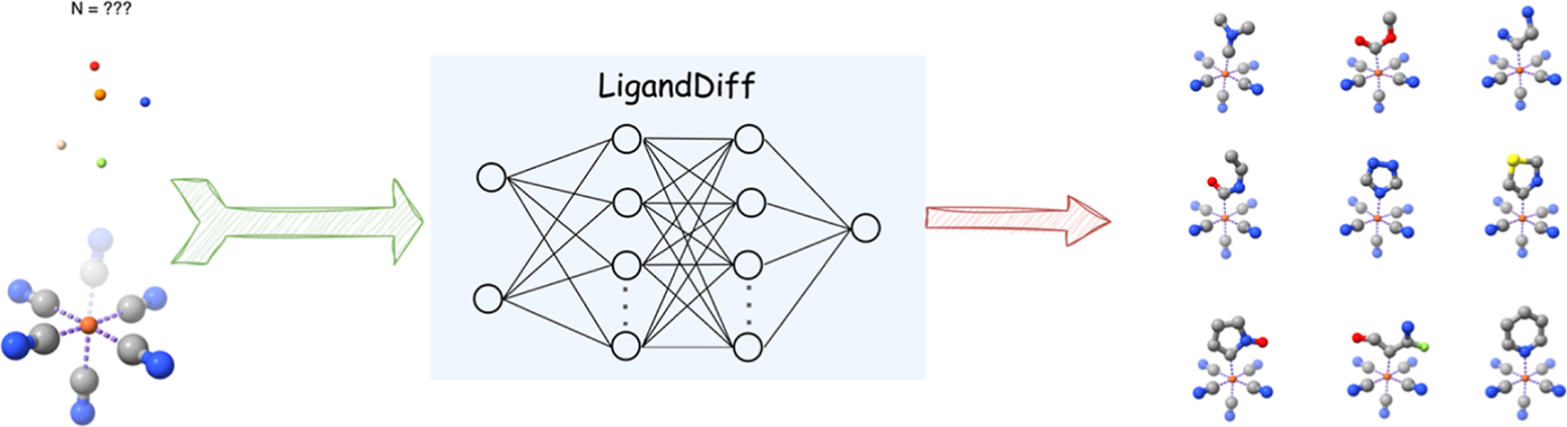

Transition metal complexes are a class of compounds with varied and versatile properties, making them of great technological importance. Their applications cover a wide range of fields, either as metallodrugs in medicine or as materials, catalysts, batteries, solar cells, *etc.* The demand for the novel design of transition metal complexes with new properties remains of great interest. However, the traditional high-throughput screening approach is inherently expensive and laborious since it depends on human expertise. Here, we present LigandDiff, a generative model for the de novo design of novel transition metal complexes. Unlike the existing methods that simply extract and combine ligands with the metal to get new complexes, LigandDiff aims at designing configurationally novel ligands from scratch, which opens new pathways for the discovery of organometallic complexes. Moreover, it overcomes the limitations of current methods, where the diversity of new complexes highly relies on the diversity of available ligands, while LigandDiff can design numerous novel ligands without human intervention. Our results indicate that LigandDiff designs unique and novel ligands under different contexts, and these generated ligands are synthetically accessible. Moreover, LigandDiff shows good transferability by generating successful ligands for any transition metal complex.

## Introduction

Molecular generation is an important tool for the discovery of new materials and drug design. It aims to create new structures with desirable properties. However, traditional methods can take a long time and are expensive. For example, the estimated expenditure for a new drug from design to production ranges from $314 million to $2.8 billion and keeps rising,^1^ and it usually takes over 12 years to develop a new drug with suitable bioavailability.^2^ It has been estimated that nearly 10^23^ to 10^60^ potential drug-like molecules are synthesizable in chemical space,^3^ wherein only 10^8^ to 10^10^ molecules have already been synthesized.^4^ It is extremely time-consuming to identify novel drugs *via* brute-force high-throughput screening, and human intuition can bias small molecule searches thereby missing novel molecules with optimal properties.

Recently, generative models have opened new pathways for molecular generation. Examples include generative models based on SMILES strings, like the variational autoencoder (VAE)-based^5^ and sequence to sequence autoencoder (seq2seq AE)-based;^6^ 3D full-molecule generative models, such as molGAN,^7^ GraphRNN,^8^ as well as scaffold-based generative models, like DeepScaffold^9^ and EMPIRE.^10^ To the best of our knowledge, however, all previous work was aimed at generating small organic molecules. The introduction of metal ions into a biological system has promising applications in clinical therapy and diagnostics.^11,12^ In 1965, Rosenberg *et al.*(13) at Michigan State University serendipitously discovered the anticancer properties of cisplatin which kick-started modern research on metallodrugs. Unlike organic drugs, metal-based agents have versatile electronic and structural properties. The flexible oxidation state of the metal enables it to coordinate with different types of ligands in various geometries. Such flexibility also offers novel reaction mechanisms, such as ligand exchange, metal/ligand-based redox activity, and photoactivation.^14^ With these unique reaction pathways, metallodrugs can easily bind with DNA or proteins at target sites to cause structural lesions, ultimately resulting in cellular apoptosis.^15,16^ Metallodrugs can also modulate the proliferation of tumor cells *via* catalyzing chemical transformations *in vivo*.^17^ In both cases, the metal is the focus of the metallodrug and tunes the 3D shape of the small organic ligands attached to the metal center. Metallodrugs open different and unique pathways for disease treatment, which traditional organic drugs cannot achieve due to drug resistance.^18^ In addition, organometallic complexes also have a wide range of applications in materials, like solar cells,^19^ electrocatalysts,^20^ batteries,^21^*etc.*

Given the great importance of organometallic complexes in both medical and industrial applications, much attention has been paid to the design of new organometallic complexes. However, current methods simply extract the already available ligands from the Cambridge Structural Database (CSD) and then combine the ligands with the metal to generate new complexes.^22,23^ This limits the investigation of novel ligand domains, which further restricts the discovery of novel organometallic complexes since once the ensemble of ligands is determined, the corresponding number of complexes is also determined. In addition, in this workflow, much work, such as ligand curation and combination, still needs human involvement and intuition, which also slows down the entire process.

In this work, we introduce LigandDiff, a scaffold-based diffusion model, to generate 3D transition metal complexes from scratch. Diffusion models^24^ are a class of probabilistic generative models which destroy the initial clean input data by progressively introducing random noise, then reverse the whole process for new sample generation. This method has been widely used in inpainting,^25^ video generation,^26^ natural language generation,^27^ 3D small organic molecule generation,^28,29^ medical image reconstruction,^30^*etc.* Denoising diffusion probabilistic models (DDPMs)^31^ are a type of diffusion model and are inspired by nonequilibrium thermodynamics. A DDPM includes two Markov chains, namely, the forward chain and the reverse chain. The forward chain keeps adding random noise, sampled from Gaussian distribution, to a clean data point *x*_0_ within predefined steps *T* to generate a sequence of noisy data points *x*_1_, *x*_2_, ···, *x*_*T*_. At a given step *t* = 0, ..., *T*, the transition kernel is defined as
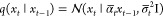
1
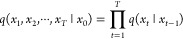
2where  determines how much information is kept, while  determines how much noise is added.^29^ Also, a diffused state *x*_*t*_ is obtained as
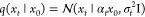
3where  = α_*t*_/α_*t*–1_ and . As proposed by Sohl-Dickstein *et al.*,^24^ σ_*t*_^2^ = 1 – α_*t*_. Usually, this noise schedule is predefined, and it smoothly transitions from α_0_ ≈ 1 toward α_*T*_ ≈ 0. Intuitively, *x*_*T*_ is pure noise without any structural information included. The denoising process or the reverse step is derived as
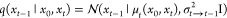
4and the mean and variance are defined as
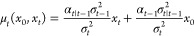
5
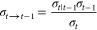
6where  and . Equation 4 indicates that any intermediate state *x*_*t*_ in this diffusion trajectory can be derived from the initial state *x*_0_ and the final state *x*_*T*_. With this property in mind, the generative denoising process starts from a prior distribution *p*(*x*_*T*_)
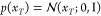
7and it aims to invert the diffusion trajectory while *x*_0_ is unknown. To achieve this, a neural network ϕ is introduced and the generative transition is then defined as
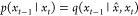
8where *x̂* = ϕ(*x*_*t*_, *t*), an estimate of *x*_0_ predicted by ϕ. Inspired by Ho *et al.*,^31^ the neural network is further adapted to predict the added noise, *i.e.*, ϵ̂_*t*_ = ϕ(*x*_*t*_, *t*). Then the estimated *x̂* is derived as
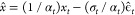
9

The object of this model is to minimize *via* mini-batch gradient descent optimization. Once this model is well trained, new sample points can be generated. Any sample point  is iteratively denoised *via*eq 8 for *t* = *T*, ..., 0 to obtain a new data point *x*_0_.

Another feature of LigandDiff is that it is scaffold-based, *i.e.*, it only diffuses or denoises one ligand while other ligands as well as the central metal are fixed at each step. In drug discovery, such “scaffold modeling” is widely used where a large portion of the molecule is kept fixed while the remaining parts of the molecule are modified.^32^ Keeping the main scaffold structure while modifying small functional groups allows for accurate and quick design of new drugs with desirable properties.^33^ Generative models can further speed up such targeted exploration of the chemical space due to their powerful flexibility with little or no human intervention. In addition, the generation of one new ligand is similar to ligand substitution, which is a useful tool for new material discovery in organometallic complexes.^34,35^ Overall, LigandDiff can be used to investigate the structure–activity relationship (SAR) in the area of metal–ligand interactions. For example, ferrocene (Fc), a “sandwich” organometallic complex, has two stable cyclopentadienyl groups which can be easily redesigned by either replacing the whole group with different organic groups or simply attaching extra functional groups to the five-membered rings, leading to a large variety of derivatives. Indeed, Fc analogues have shown potential as drug candidates against malaria as well as cancer, and each modified cyclopentadienyl moiety has specific mechanisms by which they interact with biomolecules, which improves the overall therapeutic efficacy.^36^ We anticipate that LigandDiff has the potential to accelerate the process of lead optimization for this and other classes of organometallic compounds.

## Methods

### Data Set

Arunachalam and co-workers reported a set of ∼86k mononuclear octahedral transition metal complexes,^37^ from which we curated M complexes, M = Cr, Mn, Fe, Co, Ni, Cu, and Zn with 100 atoms or less. We further constrained the nonmetal elements to {H, C, N, O, F, P, S, Cl, and Br}. Next, complexes with missing hydrogens or disorder were excluded, leading to a set of 23,308 complexes, each of which has at least two ligands. We then used molSimplify^38,39^ to break the complexes apart to obtain ligand information. Each ligand is masked for diffusion/generation, for example, for a complex with six ligands, we can obtain six variations of this complex, each of which has a unique ligand to diffuse/generate. With such an implementation, we finally obtained 87,531 samples. All hydrogen atoms were removed to reduce the computational cost. Two subsets of 400 samples were used for validation and testing, while the remaining data were used for training.

### Molecule Representation

All complexes are regarded as 3D point clouds in space. A point cloud *x* is denoted as *x* = [*r*, *h*_a_, *h*_L_], where *r* is the atom coordinates , *h*_a_ is the one-hot representations of atom type , *N* is the total number of atoms, and *m* is the number of atom types. *h*_L_ is a one-hot embedding to encode the ligand group information, *i.e.*, for a given atom, it belongs to which ligand; *h*_L_ = , where *l* is the number of ligands. In LigandDiff, before passing through the neural network ϕ, the noise is added to only the coordinates and the atom types that belong to the diffused ligand, while the ligand-group embeddings are unchanged. Although the whole atomic embeddings *x* = [*r*, *h*_a_, *h*_L_] are updated through the neural network, we consider only the predicted coordinates and the predicted atom type features.

### 3D-Conditional Diffusion Models

In LigandDiff, the assigned ligand *x*^L^ in a given complex is diffused or denoised under a fixed context *u*, *i.e.*, the undestroyed ligands as well as the central metal. The context is multifunctional. LigandDiff aims to generate realistic ligands rather than some random atoms. During the diffusion/training step, the model is able to learn what a chemically realistic ligand should be like by viewing the clean ligands in the context so that it can know how much noise is added into the diffused ligand. In addition, the context is introduced for the conditional generation. Each sample in the data set has a unique context. This uniqueness guides the model to generate a specific ligand under a specific context instead of always giving the same output, regardless of the given context. The context *u* has the same embedding constituents as *x*^L^. Under the context, the generative process in eqs 8 and 9 are adapted as
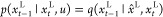
10
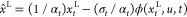
11

**Figure 1:**
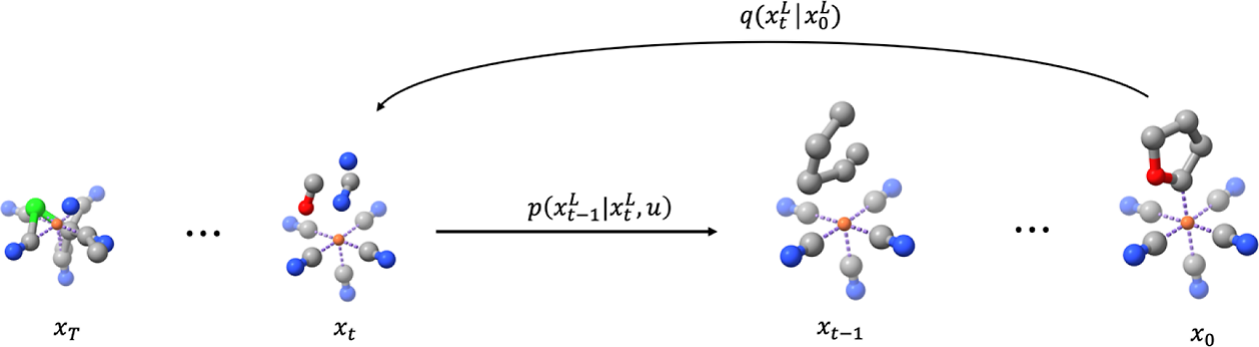
Overview of LigandDiff. It starts from the forward diffusion process *q* from *x*_0_^L^ to *x*_*t*_^L^ to sample noised data for a given ligand *x*^L^. Once the model is well trained, any new ligand can be generated from  by iteratively denoising *x*_*t*_^L^ through the conditional distributions *p*.

The schematic process of LigandDiff is given in Figure 1. During the diffusion/training step (from right to left in Figure 1), a sequence of diffused ligands with different amounts of noise is sampled in the training data set. These diffused ligands, along with their context, are then passed to a neural network ϕ to parametrize the model so that it can accurately predict how much noise is added in each diffused ligand. Once the model reaches convergence, it can be used to generate new ligands, which is the denoising/generative process (from left to right in Figure 1). The model first generates random isolated data points without any chemical indication, and it then predicts and removes the noise from these disorganized points step by step and finally generates chemically meaningful ligands.

**Figure 2:**
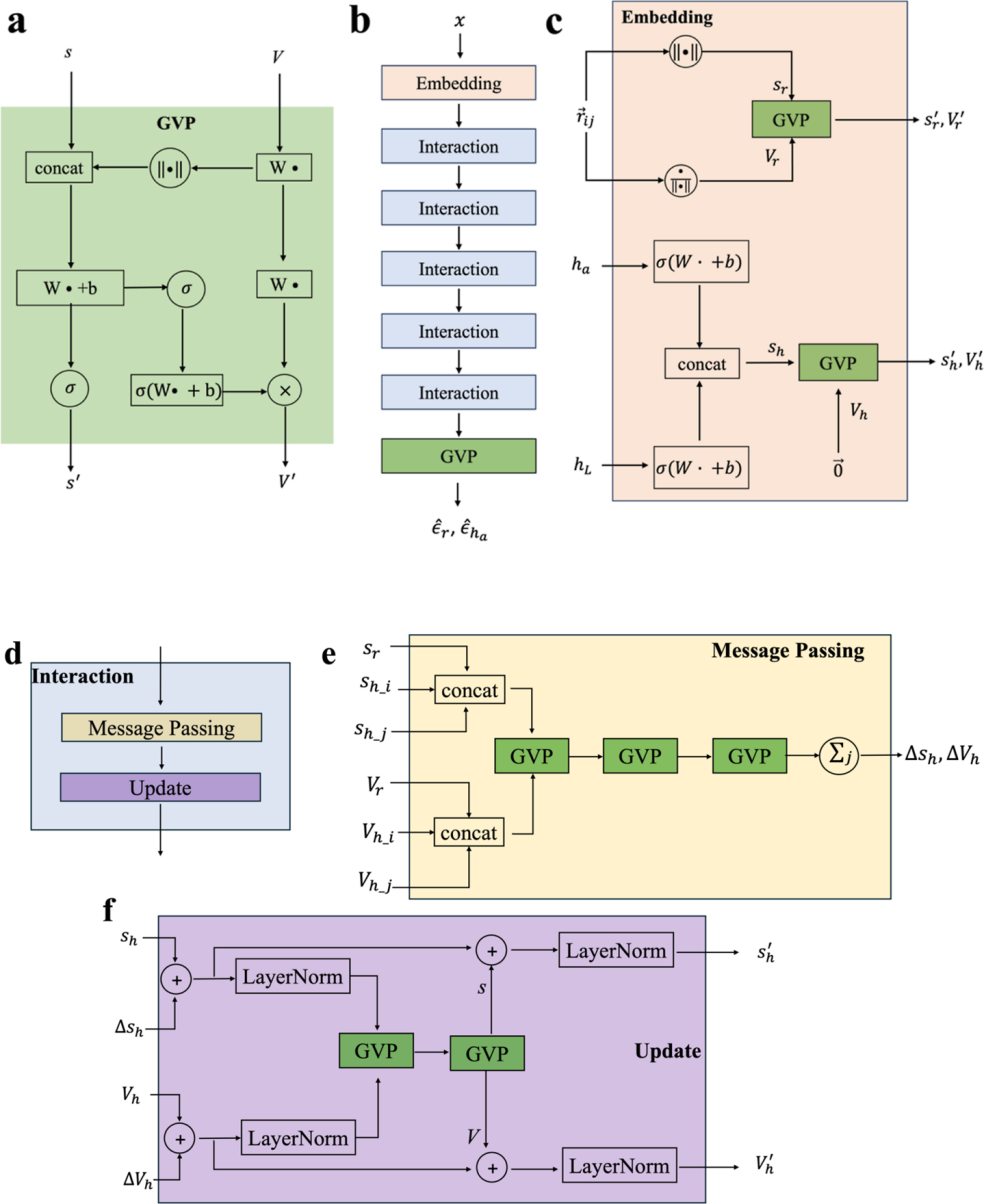
(a) GVP framework. (b) Overview of the model. (c) Embedding module. (d) Interaction module. (e) Message passing block. (f) Update block.

The noise estimation module ϕ is based on message passing neural networks (MPNNs),^40^ a framework of graph neural networks (GNNs) where each molecule is regarded as a graph and each atom is a node, while each bond is an edge. Geometric vector perceptron (GVP)^41,42^ is used throughout LigandDiff to model the molecular representation. GVP updates both scalar features, , and vector features, , simultaneously for a given tuple embedding. GVP includes two separate nonlinear transformations for the scalar and vector features. After the first linear transformation, the norm of vector features is concatenated to the scalar features to extract the invariant rotational information from vectors *V*. The mixed scalar features are then nonlinearly transformed to get the final updated scalar features *s*′. The scalar features also propagate to the vector channels by operating element-wise multiplication after another nonlinear transformation, yielding the updated vector features *V*′. The model of noise estimation in LigandDiff includes three parts: embedding, interaction, and GVP. In the embedding block, the first part is the edge information which is based on the relative position . Both initial scalar features and vector features are obtained by normalizing the . The initial edge embedding, *e* = (*s*, *V*), is further updated *via* one GVP. The scalar features of the node embedding start from the concatenation of separate nonlinear transformation of *h*_a_ and *h*_L_. The vector features are denoted as  since nodes have no directional information initially. Similarly, one GVP is utilized to update the initial node embedding, *h* = (*s*, *V*). The interaction block is used to update the node embedding and is usually repeated multiple times. It includes message passing and update blocks. In message passing, the central atom *i* aggregates information from all other atoms *j* based on the edge embedding *e*_*ij*_. The scalar edge features *s*_r_, the scalar features of the central node *s*_h_*i*_, and the scalar features of neighbor *s*_h_*j*_ are concatenated together. The vector features operate with the same concatenation. Both types of features are then passed to three GVPs sequentially to collect and then aggregate neighbors’ information, Δ*s*_h_ and Δ*V*_h_. The update block consists of two residual modules. The aggregated node information is added to the initial node embedding. After normalization, the merged information is passed to two GVPs and moves to the second residual module, which includes addition and normalization as well. The output of this interaction then enters the next interaction and iterates a couple of times. After the last GVP block, the noise of atom position ϵ̂_r_ and element type ϵ̂_h_a__ is determined. A schematic depiction of the whole model is given in Figure 2.

### Training

LigandDiff was trained with *T* = 500 diffusion steps. The model has 5 layers with 192 hidden features with a batch size of 64. It uses the Adam optimizer with the learning rate of 1.0 × 10^–4^. The model was trained on a single NVIDIA A100 GPU, and it took about 7 min for one epoch.

### Ligand Generation

Once the model is well trained, users can generate numerous ligands with their own complexes. The generation process is easy to start. Users only need to specify the size of the generated ligands and the number of generated ligands. For example, given a complex with 6 ligands, if a user wants to generate 10 new ligands for each coordination site, LigandDiff finally generates 60 variations of the given complex, each of which has a different ligand from the reference complex. Moreover, this generation process is very efficient. For our test set, which includes 400 samples, LigandDiff takes ∼3 min to generate 400 ligands where we only generate one ligand for each sample in our test set.

### Assessment Metrics

Various metrics are used to fully assess the performance of LigandDiff. We first use OpenBabel^43^ to add bonds to the generated data points *x*^L^. Then the validity of the generated ligands is evaluated by RDKit.^44^ The metric of the model’s ability to generate valid ligands is given as
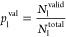
12where *N*_l_^valid^ is the number of valid ligands, and *N*_l_^total^ is the number of total ligands generated. The connectivity of the ligands is used to check whether all atoms in the valid ligands are fully connected, which is calculated as
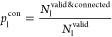
13where  is the number of valid and connected ligands. The uniqueness and novelty are also evaluated as
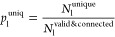
14
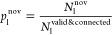
15where  is the number of unique ligands among outputs, and  is the number of the ligands outside the training data set. For both metrics, all valid and connected ligands are converted to SMILES for evaluation. Ligands with identical SMILES numbers are considered duplicates. Finally, we assess the validity of the whole complex using molSimplify to check whether there exists molecular overlap, calculated as
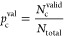
16where *N*_c_^valid^ is the number of valid complexes.

## Results and Discussion

### Random Sample

In the test set, 105 out of 400 samples have only one heavy atom as a ligand to generate, and half of the complexes have less than 5 heavy atoms to generate. We believe that it is easy for LigandDiff to sample valid complexes under such conditions. To strictly evaluate the performance of LigandDiff, we randomly sample the size of the generated ligands in the range 6 to 20. This range is chosen because it only covers 41.7% of the size distribution in the training data set, while 51.5% of the diffused ligands in the training set have 5 heavy atoms or less. The results are reported in Table 1. Although this size sample is challenging for LigandDiff, it still shows remarkable performance. Unlike other generative models,^45,46^ which explicitly employ valency rules to improve validity, LigandDiff is able to learn these rules implicitly and thus generates what are perceived as valid ligands. Moreover, these valid ligands are highly connected and unique, leading to 90% valid complexes.

**Table 1:** Performance of LigandDiff with Regard to Validity, Connectivity, Uniqueness, and Novelty^a^

	*N*_atom_	*p*_l_^val^	*p*_l_^con^	*p*_l_^uniq^	*p*_l_^nov^	*p*_c_^val^
random sample	6–20	0.94 ± 0.012	0.96 ± 0.008	0.97 ± 0.009	0.96 ± 0.009	0.90 ± 0.016
fix the ligand size	6	0.97 ± 0.006	0.94 ± 0.012	0.56 ± 0.013	0.81 ± 0.016	0.91 ± 0.015
	7	0.97 ± 0.009	0.95 ± 0.015	0.70 ± 0.094	0.83 ± 0.018	0.92 ± 0.014
	8	0.97 ± 0.007	0.95 ± 0.005	0.89 ± 0.018	0.94 ± 0.011	0.91 ± 0.011
	9	0.96 ± 0.011	0.95 ± 0.007	0.90 ± 0.012	0.98 ± 0.007	0.90 ± 0.014
	10	0.96 ± 0.012	0.95 ± 0.008	0.92 ± 0.030	0.98 ± 0.004	0.91 ± 0.010
	11	0.96 ± 0.008	0.96 ± 0.010	0.95 ± 0.009	0.99 ± 0.007	0.91 ± 0.014
PPR_100	11–40	0.94 ± 0.017	0.94 ± 0.015	0.92 ± 0.019	1.0 ± 0	0.87 ± 0.026

a The results are reported as “mean ± std” over 10 independent runs.

### Fixing the Ligand Size

**Figure 3:**
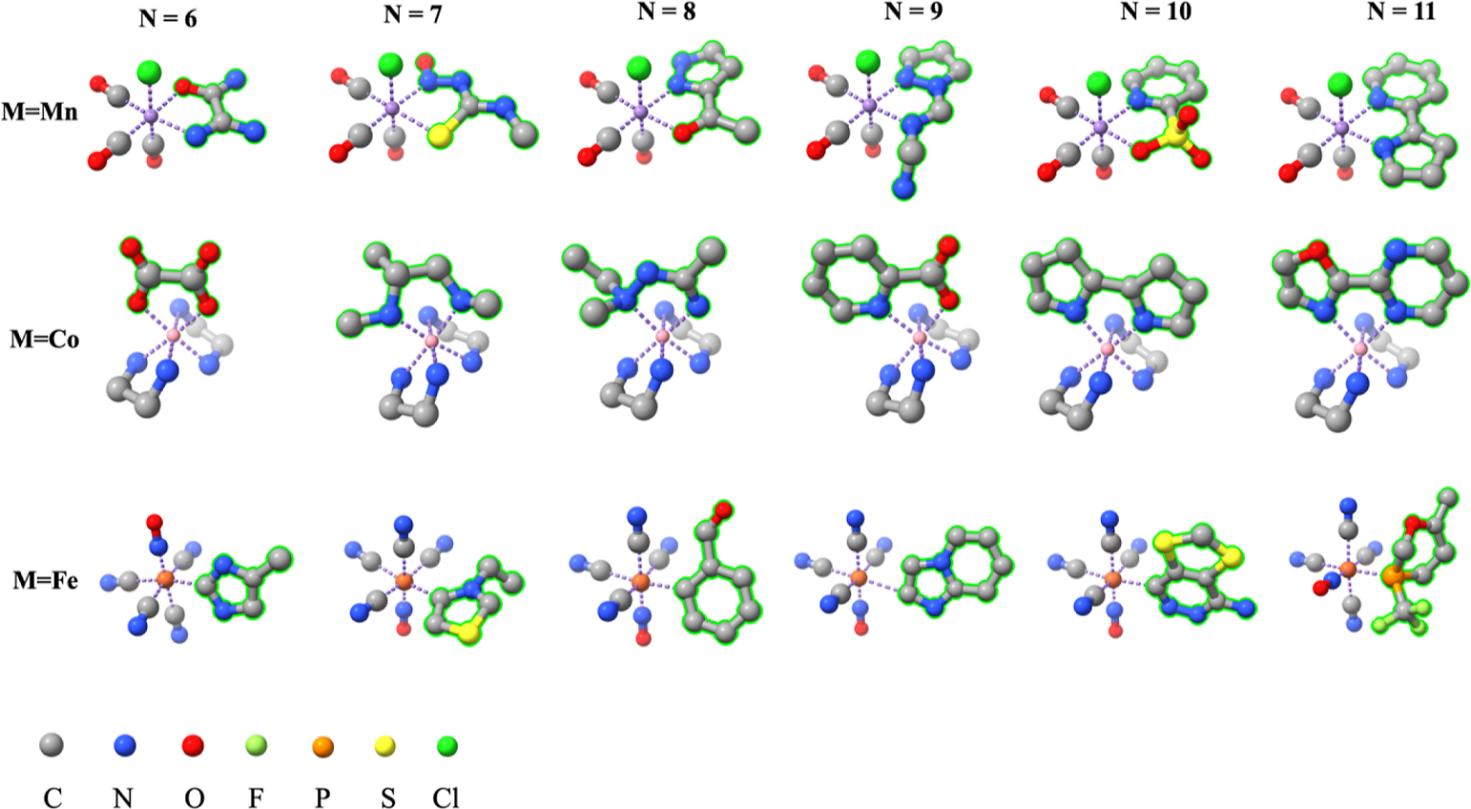
Generated complexes under a fixed ligand size. Each column has the ligand with the identical size to generate but under a different context, while each row has the ligand with an increasing size to sample under the same context. The generated ligand is highlighted in green outline.

To assess whether the model learns chemical principles or just memorizes the ligands in the training set, we fixed the size of the generated ligand, *i.e.*, each complex in the test set now has a ligand with the same size to generate. We start from the ligand size *n* = 6 and increase it to 11. As shown in Table 1, the metrics of validity are at a consistently high level, where the validity of the ligands and the whole complexes are around 0.96 and 0.91, respectively. In addition, the connectivity is also noteworthy, yielding more than 94% connected ligands. The rapid increase in uniqueness and novelty strongly indicates that LigandDiff “learns chemistry with respect to bonding patterns” to some extent and can use this knowledge to generate new and valid ligands. For *n* = 6, uniqueness is only 0.56, which means nearly half of the successfully generated ligands are duplicates (same configuration but in different conformations), while this metric reaches 0.95 when n increases to 11. The observed improvement is consistent with our understanding of chemistry, *i.e.*, the diversity of structures increases as the size of the system increases. Also, LigandDiff appears to understand this and keeps generating different and diverse ligands. This also applies to novelty. Instead of simply generating the ligands which already exist in the training set, LigandDiff tends to design completely new ligands. Even with *n* = 6, LigandDiff still can generate 81% novel ligands and when *n* reaches 11, the generated ligands are almost exclusively outside the training set. All these results show that in this extreme situation where all the generated ligands have to have the same size, LigandDiff still can generate different ligands under the given context. Some examples are given in Figure 3.

### Beyond Cr, Mn, Fe, Co, Ni, Cu, and Zn

**Figure 4:**
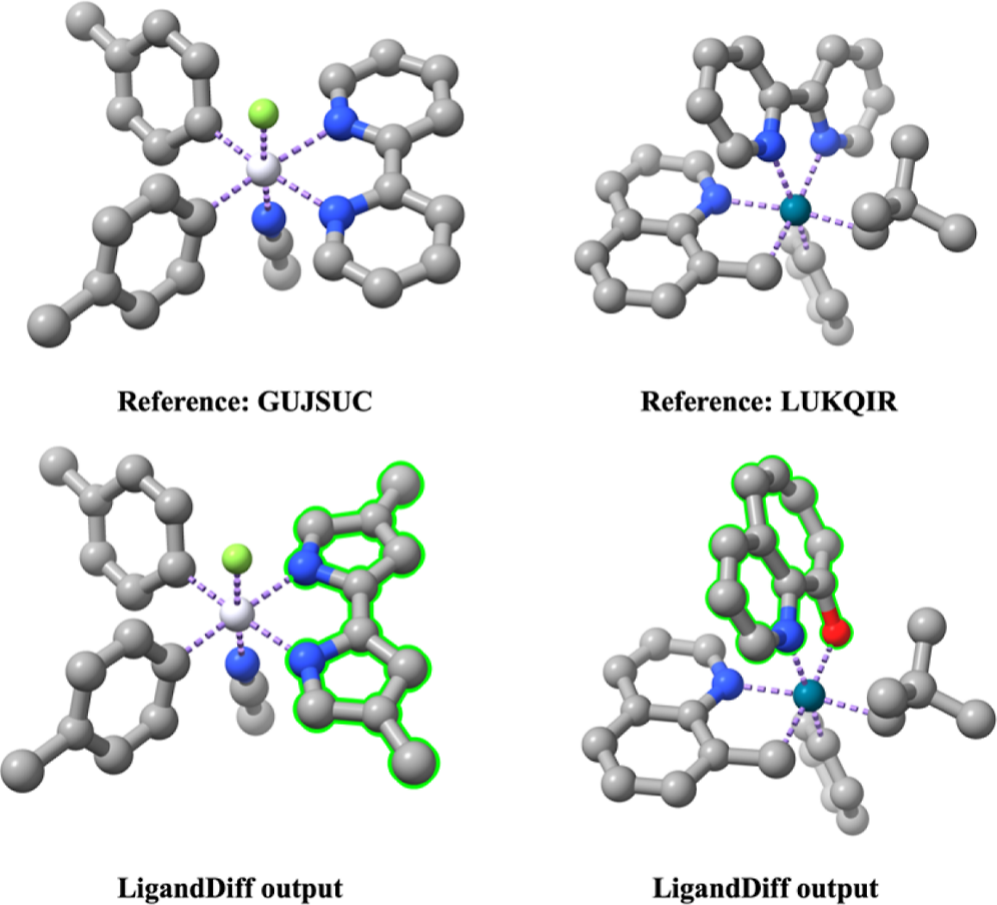
Examples of generated complexes (bottom) and the corresponding reference complexes (top) in the PPR_100 set. The CSD code is given.

To further assess the capability of LigandDiff, we curated a challenging data set termed PPR_100 from the original database.^37^ The PPR_100 set includes 100 Pt, Pd, and Ru complexes with more than 50 atoms. These three types of transition metal complexes were chosen since they are the top 3 complexes in the database, except for the transition metal complexes already covered in our data set. For each complex, we mask the ligand with more than 10 heavy atoms to generate a new ligand with the same size, and 148 samples are obtained because some complexes have one more suitable ligand to mask. Since 67.8% of the diffused ligands in the training set have 10 or fewer heavy atoms, this becomes a challenge for LigandDiff to generate valid complexes. The results are given in Table 1. Although Pt, Pd, and Ru are not included in the training set, LigandDiff is still able to generate 94% valid and connected ligands with 100% novelty. Again, LigandDiff generates these ligands with high diversity since only 8% of ligands are duplicates and 87% complexes are valid as indicated by molSimplify. Some examples of the generated complexes as well as the reference complexes are shown in Figure 4. In LigandDiff, the metal is constrained in the context, and it is only used to predict the noise of the generated ligand, but the metal itself is never involved in the diffusion process. Such a flexible design enables LigandDiff to generate novel ligands for any transition metal.

### Synthetic Accessibility

To assess whether the generated ligands are synthesizable, we calculate the average synthetic accessibility (SA) score.^47^ As shown in Table 2, LigandDiff generates realistic ligands with high SA. This capability remains at a high level as the ligand size increases.

**Table 2:** SA Scores of Ligands Generated by LigandDiff

	*N*_atom_	SA^a^
random sample	6–20	0.69 ± 0.008
fix the ligand size	6	0.80 ± 0.005
	7	0.77 ± 0.020
	8	0.74 ± 0.005
	9	0.74 ± 0.003
	10	0.73 ± 0.008
	11	0.72 ± 0.008
PPR_100	11–40	0.68 ± 0.008

a 0 = hard, 1 = easy.

## Conclusions

The design of novel organometallic complexes is highly demanding but worth the effort given their various applications. In this study, we described LigandDiff, a 3D-conditional diffusion model for transition metal complex generation. LigandDiff designs realistic ligands under a set of given ligands and their associated metals, and it is capable of generating novel and unique ligands which is relevant for molecular design. Moreover, we found LigandDiff to be transferable and can design ligands for transition metals that are not included in the training data set. Overall, we believe this tool has the potential to facilitate the design of novel organometallics for applications ranging from metallodrugs to materials.

## Data Availability

All data and code are available at https://github.com/Neon8988/LigandDiff.

## Supporting Information Available

Size distributions of the diffused ligands in training data set and synthetic accessibility calculation (PDF)
