# Supplementary material for: LigandDiff: de Novo Ligand Design for 3D Transition Metal Complexes with Diffusion Models
Source: J Chem Theory Comput. 2024 May 14;20(10):4377–84. doi: 10.1021/acs.jctc.4c00232 (PMC11137811; doi:10.1021/acs.jctc.4c00232)
Supplement: Supplementary file 1 — ct4c00232_si_001.pdf [file ct4c00232_si_001.pdf]

Supporting Information for LigandDiff: de Novo Ligand Design for 3D Transition Metal Complexes with Diffusion Models

Hongni Jin[a] and Kenneth M. Merz, Jr.[a,b]*

[a]Department of Chemistry, Michigan State University,

East Lansing, Michigan 48824, United States

[b]Department of Biochemistry and Molecular Biology, Michigan State University,

East Lansing, Michigan 48824, United States

*Email: merz@chemistry.msu.edu

The size distributions of diffused ligands in the training dataset

Table S1 lists the size distributions of the diffused ligands for the forward process. More than half of the diffused ligands have 5 heavy atoms or less.

Table S1. The diffused ligands in the training dataset

size	Counts
1~5	44665
6~20	35884
21~40	5992
41~60	184
61~80	6

Synthetic accessibility score

In the original work,[1] the synthetic accessibility (SA) is ranked from 1 to 10 and molecules with a high SA score is evaluated as hard to synthesize. To better interpret the results, we further normalize the SA score into the rang of 0 ~1, calculated as

$$SA_{norm} = \frac{10 - SA}{9}$$

Reference

(1) Ertl, P.; Schuffenhauer, A. Estimation of Synthetic Accessibility Score of Drug-like Molecules Based on Molecular Complexity and Fragment Contributions. *J Cheminform* **2009**, *1*, 8.
